# Rhabdomyolysis from Resistance Exercise and Caffeine Intake

**Published:** 2018-01

**Authors:** Dong Jun SUNG, Eun-Ju CHOI, Sojung KIM, Jooyoung KIM

**Affiliations:** 1. Division of Sports Science, Konkuk University, Choong Ju, Korea; 2. Dept. of Physical Education, Catholic University of Daegu, Gyeongsan, Korea; 3. Dept. of Physical Therapy, University of Massachusetts, Lowell, Massachusetts, USA; 4. Health and Rehabilitation Major, Kookmin University, Seoul, Korea

## Dear Editor-in-Chief

Rhabdomyolysis is a pathological condition of skeletal muscle damage (1) that can lead to various complications including acute renal failure, and compartment syndrome (2). The contributing causes of exercise-induced rhabdomyolysis are suggested to be excessive overload exercise, overdosing on certain supplements and drugs, nutritional factors, being vegetarians, and exercising in hot environments (3). We reported on the observation of rhabdomyolysis for a 29-yr-old healthy woman performed usual regular exercise and had consumed ephedra-containing herbal medicine for weight loss (1). The implications were that even for people regularly exercising, they could be at a risk for rhabdomyolysis when taking certain drugs, being on certain diets, and their lifestyle.

In this regard, the intake of coffee or caffeine-containing drinks has increased recently, as it may be attractive for those who participate in exercise for weight loss to know that caffeine intake before or during the exercise induces an increase in the lipolysis (4) in skeletal muscle cells. However, such caffeine intake may increase the risk of rhabdomyolysis. Looking into supportive evidence, a 44-yr-old woman admitted to hospital due to coffee-colored urine 6 h after excessive intake of black coffee (4 cups, approximately 100 ml per cup) had the finding of rhabdomyolysis, which was thought to be contributed by excessive caffeine intake (5). There was also a case of a male who took in 24 g of caffeine and was observed to have rhabdomyolysis and acute renal failure (6). Thus, caffeine (ingested alone or in combination with exercise) is recognized as a potential risk factor for rhabdomyolysis. The mechanistic link may be as caffeine increases intracellular Ca^2+^ concentrations by binding and activating the inositol trisphosphate (IP3) receptor (7), and thus amplifying a variety Ca^2+^ increase pathways (store-operated Ca^2+^ entry, Ca^2+^-induced Ca^2+^ release), which over time can lead to damaging the sarcoplasm (3). This may, in turn, harm the muscle cell and result in rhabdomyolysis. Therefore, we would like to report a case of rhabdomyolysis where there was a combination of intake of caffeine-containing drinks and exercise.

Written informed consent was obtained from the patient for publication.

In this study, a 21-yr-old male (height=177 cm, weight=73.7 kg) without current disease, previous illnesses or a family history conducted resistance training on a regular basis (at least 4 d/week, 1 h) for a year or more. The patient also drank 3 large cups of S’s coffee (454 mL per cup, approximately 1362 mL for three cups, approximately 150 mg of caffeine per cup, approximately 450 mg of caffeine for three cups) 30 min prior to conducting his usual exercise routine. He was admitted to the emergency room, complaining of an unusual thigh muscle pain and having a brown-colored urine approximately 30 h after exercise. He was subsequently diagnosed with rhabdomyolysis. In the interview, the patient mentioned the unusually large intake of coffee before the onset of symptoms and before his exercise routine. He noted, however, that the temperature in the gym and the exercise program were as usual. In addition, the patient showed a relatively high sensitivity to caffeine, as the symptoms such as sleep disorder and tension increases coincided with coffee intake. A heart rate of 77 beats/min with normal electrocardiography was observed on early diagnosis but 16500 U/L (reference 39–308) of creatine kinase and 725.9 ng/mL (reference 0–25) of myoglobin were observed on the blood test. In addition, related to liver function, 972 U/L (reference 0–40) of aspartate transaminase and 237 U/L (reference 0–41) of alanine transaminase were observed. Possible complications were not present (Table 1).

**Table 1:** Blood test values in the patient with rhabdomyolysis

***Test***	***Value***	***Reference***
AST (U/L)	972	0–40
ALT (U/L)	237	0–41
MB (ng/mL)	725.9	0–25
CK (U/L)	16500	39–308
CK-MB (ng/mL)	159	0.6–6.3
LDH (U/L)	2796	132–225

Abbreviations: AST = aspartate transaminase; ALT = alanine transaminase; MB = myoglobin; CK = creatine kinase; LDH = lactate dehydrogenase.

Even if the subject conducts regular exercise, intake of caffeine-containing drink may be a risk factor of potential rhabdomyolysis. Although daily caffeine doses that could lead to rhabdomyolysis in previous studies were mostly in gram quantities (1.45g–17.5 g) (8, 9), the individual’s sensitivity to caffeine should be considered. A person who has a relatively high caffeine sensitivity or combines exercise and high caffeine intake for weight loss should be made aware of the risks for rhabdomyolysis and its early symptoms and should seek prompt treatment before worsening of the symptoms.
